# Secure and Efficient Key Coordination Algorithm for Line Topology Network Maintenance for Use in Maritime Wireless Sensor Networks

**DOI:** 10.3390/s16122204

**Published:** 2016-12-21

**Authors:** Walid Elgenaidi, Thomas Newe, Eoin O’Connell, Daniel Toal, Gerard Dooly

**Affiliations:** 1 Optical Fibre Sensors Research Centre, Department of Electronic and Computer Engineering, University of Limerick, Limerick V94 T9PX, Ireland; walid.elgenaidi@ul.ie (W.E.); eoin.oconnell@ul.ie (E.O.); gerard.dooly@ul.ie (G.D.); 2 Mobile and Marine Robotics Research Centre, Department of Electronic and Computer Engineering, University of Limerick, Limerick V94 T9PX, Ireland; daniel.toal@ul.ie

**Keywords:** wireless sensor networks, WSN, security, maritime WSN, dynamic symmetric key update, Waspmote, algorithm

## Abstract

There has been a significant increase in the proliferation and implementation of Wireless Sensor Networks (WSNs) in different disciplines, including the monitoring of maritime environments, healthcare systems, and industrial sectors. It has now become critical to address the security issues of data communication while considering sensor node constraints. There are many proposed schemes, including the scheme being proposed in this paper, to ensure that there is a high level of security in WSNs. This paper presents a symmetric security scheme for a maritime coastal environment monitoring WSN. The scheme provides security for travelling packets via individually encrypted links between authenticated neighbors, thus avoiding a reiteration of a global rekeying process. Furthermore, this scheme proposes a dynamic update key based on a trusted node configuration, called a leader node, which works as a trusted third party. The technique has been implemented in real time on a Waspmote test bed sensor platform and the results from both field testing and indoor bench testing environments are discussed in this paper.

## 1. Introduction

Recent advances in Wireless Sensor Networks (WSNs) have meant that sensor nodes are capable of processing and transmitting environmental monitored data in real time to end users who are located far from the area covered by the sensor network. WSNs are still limited however in terms of energy, memory storage, and security communication capabilities. Fortunately, security for WSNs has been examined and developed upon for different application domains, such as medical and environmental. It has been shown by Mathur et al. in [1] that it is possible to provide a patient monitoring system that resolves security issues associated with data loss, while in [2] Elgenaidi et al. have studied different water environment monitoring systems based on WSNs that carry information that has value, and this value (data) must be encrypted for protection. The security of transmitted data is crucial in WSN applications so as not to reveal to unauthorized persons the information travelling between nodes, however this security solution must be resource-friendly and efficient. In order to build an efficient security algorithm, it is necessary to fully understand the process of security functions in terms of energy consumption, time execution and code size.

In WSN mesh networks, symmetric encryption algorithms have been widely used because of the advantages of low cost with respect to power consumption, time execution and code size. The main obstruction in the implementation of symmetric encryption is the issue of key re-distribution between nodes in the case of a change in the network members. Asymmetric encryption techniques, such as Rivest, Shamir, and Adleman (RSA) and elliptic curve cryptography (ECC) have been used to tackle re-keying in WSNs applications [3], however, the problem with using these techniques is the computation overhead on every node in the network, and this increases the cost of each node and accordingly the processing of security key management. Generally, key management in sensor networks can be listed under three headings:

- Key Transport/Distribution where one sensor node creates and securely transfers it to the others.
- Key Agreement where the key is established by a shared secret between two or more nodes.
- Key pre-distribution where keys are distributed before the sensor nodes are deployed. Moreover, key management can be performed using trusted third party devices such as trusted servers, authentication servers, key distribution centers (KDCs) key translation centers (KTCs), and certification authorities (CAs) [4,5,6].

Some applications rely upon a secure key establishment mechanism where each node in a key establishment protocol is able to determine the true identity of the other nodes that could possibly gain access to the resulting key. This implies the preclusion of any unauthorized additional parties from deducing the same key [5]. Many secure key management schemes which promote longer battery life also depend on the type of system.

The most widely used wireless standard for WSNs is IEEE 802.15.4 [7]. The main advantages of using IEEE 802.15.4 are very low energy consumption, the capability of using different network topologies, for instance point-to-point topology the capability of inter-operability with transmit/receive chips, such as XBee 802.15.4 pro and the ability to engage with Wi-Fi.

The scheme being proposed in this paper addresses the main issues in security mechanisms based on the symmetric encryption algorithm, including memory, storage space, key generation, and re-keying. In this paper, the deployment of a Waspmote sensor node integrated with the XBee 802.15.4 pro module in an outdoor environment using IEEE 802.15.4/2.4 GHz standard will be used to demonstrate the implementation of the proposed protocol. Most of the presented results to date by other authors [8,9] have been obtained using simulators and not real deployments as in this paper. This paper also presents a security mechanism suitable for marine coastal monitoring, where each node in a line topology sends packets encrypted with its secret key, called the Adjacent key, to provide data confidentiality. This key is shared only with an authorized neighbor in the network. Furthermore, the re-keying phase in the revocation process will be a partly operation coordinated by a node called the leader node.

The rest of this paper is organized as follows: related works are discussed in Section 2. In Section 3 the proposed technique is discussed covering network topology, platform, travelling packets, packet structure, and security and key management. It also describes transmission security and data encryption, Re-keying and the memory requirement for the scheme. In Section 4, the outdoor implementation and performance measurements of the scheme are also presented in different scenarios. These measurements are discussed in terms of the received single strength indicator (RSSI), the average round trip time (RTT) and current consumption. In Section 5 a discussion and comparison between measurements of the proposed scheme and results of other schemes is given. The paper is concluded in Section 6.

## 2. Related Work

There are some well-known practices for developing efficient trust security systems for WSNs and managing the cryptographic keys in order to protect WSNs from malicious attacks [10]. Liu, et al. proposed in [11] an efficient and simple technique for detecting selective forwarding attacks and recovering the failed route based on a per-hop acknowledgement. This section will present key management and encryption schemes, which ensure the level of security in WSNs application.

Liebeherr et al. [12] designed and implemented a security key management and encryption scheme called the ‘neighborhood key’ scheme. This technique provided integrity and confidentiality for application data in overlay networks. The core mechanism of this technique was to avoid network-wide re-keying operations. Additionally, the scheme re-encrypts the payload data at each forwarding hop. Moreover, the neighborhood key method provided a solution for protection against routing attacks, where authentication between sensor nodes in the network depends on the certificate signed by a trusted third party using an X.509 Version 3 certificate. Each sensor node in the network had its own signed certificate, also each node stores the certificates of one or more trusted third party. In this scheme the authentication phase was performed without coordination with other nodes. The node certificate included a secret key, which is used to encrypt or sign data. Sensor nodes exchange certificates after receiving a message protocol from another node in the network. Once the certificates are exchanged, the encryption of data and the signing of hashes in each node will be done with a single symmetric key called a ‘neighborhood key’. Thus, the neighborhood keys are shared between current authenticated neighbors in the network. In the joining phase where a new node joins the network, a new neighborhood key must be generated and sent to all of its authenticated neighbors in order to maintain confidentiality in the network.

Furthermore, updating and exchanging a new neighborhood key is executed whenever the set of authenticated neighbors are changed or the specified maximum lifetime of the current neighborhood key is expired. Therefore, every sensor node must encrypt the new neighborhood key with the public keys of all the authenticated neighbors (using the RSA algorithm), which are stored in the node during the authentication stage. The security issues are exacerbated during failures in re-establishment of the network topology when one or more nodes join/leave the network at the same time. Additionally, by implementing an integrity test and limiting the allowed frequency of transmitted key request messages the neighborhood scheme protects nodes from Denial of Service (DoS) attacks from malicious adversaries.

A hierarchical key management scheme for secure group communications in a mobile ad hoc network is proposed by Wang et al. [13] and Annadurai [14]. In this proposed scheme, a new approach with a two-layer structure where a cluster head manages information between sensor nodes in the layers was given. The main idea in this scheme is that nodes are divided into two subgroup levels, a Level 1 subgroup ‘L1-subgroup’ contains all sensor nodes in the subgroup. Moreover, Level 2 subgroup ‘L2-subgroup’ is located depending on positional information of nodes in Level 1. In order to manage data transmission and coordinate security keys between nodes in the same subgroup level and with nodes in the other subgroup level, an election of a cluster head in each level must be processed. Generally, the election of a cluster head in each level depends on the largest weight value of nodes [4]. In each L1-subgroup’, the node with the largest weight value in every L1-subgroup will be selected as the level 1 cluster head ‘L1-head’. Then to manage communication between levels and subgroups, the largest node weight value in L2-subgroup will be selected as Level 2 cluster head ‘L2-head’.

The nodes in the subgroups use the Diffie–Hellman (DH) scheme for secure transmission of their own subgroup keys, where each subgroup has a unique subgroup key [13,14]. Packets are transmitted between subgroups through the cluster heads. The L1-head generates a communication key which is shared between the different subgroups. However, the encryption and decryption operation during data transmission in different subgroups is only through subgroup keys. Furthermore, the Level 2 cluster head, ‘L2-head’, is responsible for a new node joining its subgroup.

Jang et al. [15] proposed a time-based management protocol for WSNs to establish pair-wise keys. This technique relies on probabilistic time intervals and multiple initial keys, K_I_. In this scheme, a pool of initial keys is assigned to time slots during the key setup phase. Sensor nodes are preloaded with initial key and master keys of randomly chosen time slots before the deployment phase. In the initial key establishment phase, all sensor nodes that contain K_I_ can compute a master key and then establish pair-wise keys with their neighbor node that was deployed at the same time slot using the same initial key. However, sensor nodes that were deployed at different time slots can establish pair-wise keys, if they have the same master key derived from the current initial key.

## 3. Proposed Scheme

Node configuration and security key management are fundamental characteristics to improve the performance of secure data transmission in a WSN. These security considerations require practical and accurate key management techniques. Location-based techniques solve the topology construction issues, where every node in a network knows its own position in the network. Furthermore, neighbor-based techniques make WSNs efficient in terms of packet travelling, power consumption and topology control.

### 3.1. Network Topology

**Figure 1:**
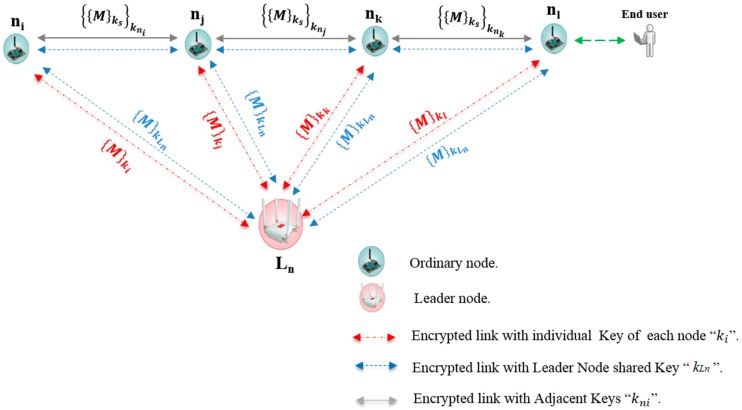
Nodes in line topology.

In most WSNs, a node needs to know some very important features, such as its own location and that of the destination node. Normally, nodes in WSNs use a location service mechanism such as the Global Positioning System (GPS) to perform the routing function [16]. In this section, an approach to manage and maintain a line topology network that is appropriate for coastal marine applications based on WSNs is presented. The scheme addresses some of the obstacles to security in WSNs, such as memory storage, communication overhead, energy consumption and the re-keying process. This mechanism relies upon the concepts of location-based routing [17]. Packets travel between nodes based on the information of the next repeater node. Subsequently, the static position of the sensor node encourages proposing a strategy for managing and controlling the transmission of secure packets between wireless nodes. In addition, maintaining network connectivity when removing or joining nodes makes it necessary to know the identification of the neighboring node/nodes in order to exchange the cryptographic keys and create a secure communication link. The main idea behind this work is to allow an ordinary node to determine its authenticated neighbor without the use of complex computations. Nodes will make their decision depending on the recommendation message from the node called the leader node (*L_n_*). The *L_n_* is located at a calculated distance from the line topology of ordinary nodes. Figure 1 illustrates the deployment of the nodes in our scheme and Table 1 provides details on the notation used.

### 3.2. The Platform

**Figure 2:**
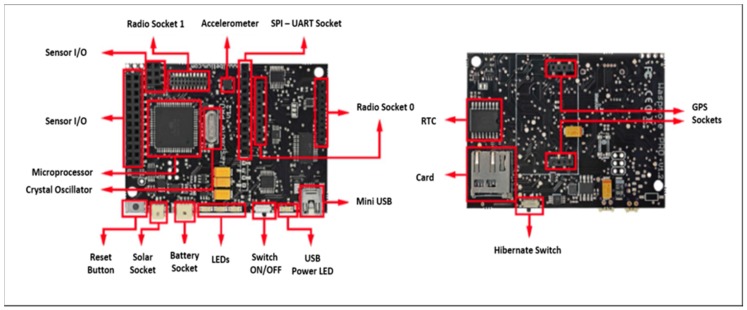
Waspmote components, top and bottom sides.

The main focus of this work is a real time testbed implementation of a secure data routing algorithm. A Waspmote platform created by Libelium (Zaragoza, Spain) as shown in Figure 2 was used. The Waspmote sensor node is provided with different frequency radio and protocols as shown in Table 2.

In the work presented here the XBee-Pro protocol is used for communications between nodes. This provides for a maximum communication distance of 7000 m between nodes which is ideal for the line topology used in this work.

### 3.3. Travelling Packets

As shown in Figure 1, the packet travels from the source to the destination based on the location of the nodes in the neighboring node’s list of members, which is coordinated from/by the leader node, *L_n_*. Each ordinary node must forward the packet to its next authenticated neighbor through link encryption with its adjacent key, ‘*k_nj_*’. The fundamental motivation behind this strategy is to configure a network line topology with simple and scalable security algorithms.

### 3.4. Packet Structure

**Figure 3:**
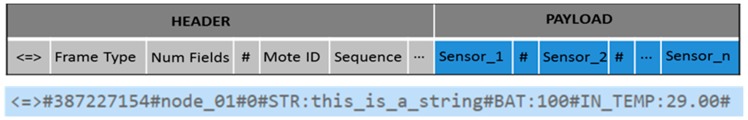
ASCII Frame structure.

**Figure 4:**
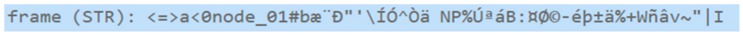
Format of the encrypted frame.

Generally, a packet consists of two different parts, namely, the header field and the payload field. The header part is a set of bytes that are usually used to determine packet characteristics, for instance, Start Delimiter, Frame Type ‘Binary/ASCII’, Node ID and Frame Sequence. The frame payload part is used to store sensor data. Figure 3 shows the ASCII frame structure of the Libelium Waspmote that is used in this work. After applying the encryption function, the Waspmote Frame encrypted with the Advanced Encryption Standard (AES) key is specified as input. This encrypted information becomes the payload of the new encapsulated frame as in Figure 4.

Although, the maximum default frame size in the Waspmote is 150 bytes per frame, the frame size depends on three characteristics:

- Encryption
- Type of XBee module used
- Transmission mode

Table 3 illustrates the maximum Waspmote frame size per protocol. The XBee 802.15.4 Link Encrypted of 16 bit Unicast and Broadcast protocols are used in this work with maximum frame size of 98 Bytes and 95 Bytes respectively (highlighted in Table 3).

### 3.5. Security and Key Management

This section presents an overview of the services provided, such as confidentiality, availability and localization, as well as a smart technique to transmit security data between sensor nodes while addressing the issues of key management. The technique presented here uses a symmetric cryptographic algorithm to encrypt the links between the ordinary nodes and the leader node. The approach that was implemented for this work was based on the Advanced Encryption Standard (AES) with a key length of 128 bits. Here AES encrypts a block of elements using the electronic codebook (ECB) encryption mode as shown in Table 4.

#### 3.5.1. Transmission Security and Data Encryption

**Figure 5:**
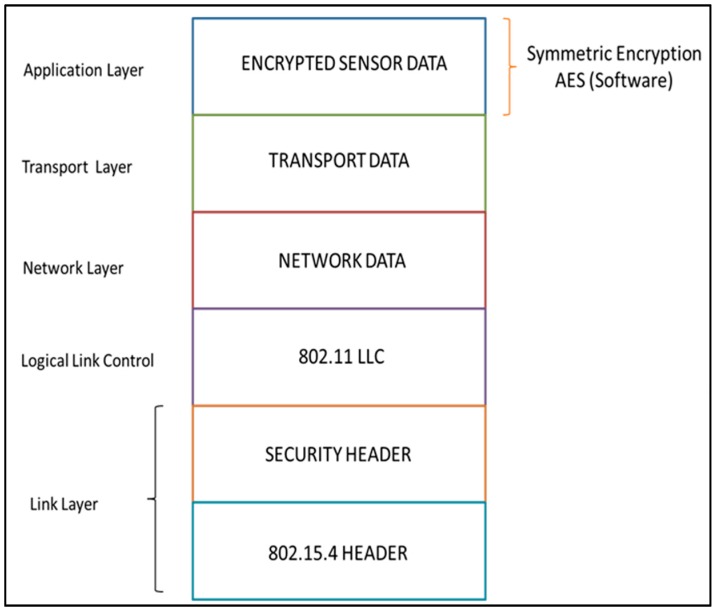
Waspmote Frame on OSI stack for communication.

As shown in Figure 5, sensor data, ‘*M*’, is encrypted in the application layer via software with AES 128 using the source key, ‘$k_{s}$’, which is shared exclusively between the source and the destination nodes.

Then, the encrypted frame is encrypted again with the shared adjacent key, ‘$k_{ni}$’ (AES-128), which is shared exclusively between every set of two neighbors as in Equation (1) below. The repeater node that forwards the sensor data to the destination in the network will decrypt the information once using the shared adjacent key, ‘$k_{ni}$’. Then, to ensure complete confidentiality and privacy, before forwarding the data to the next repeater, the node will encrypt it via its adjacent key, ‘$k_{nj}$’. Thus, the repeater will not be able to see the original sensor data transmitted due to the encryption with the source key, ‘$k_{s}$’. Equation (1) below shows this process where decryption with a shared adjacent key which is shared with the neighbor of the node performing the encryption:

(1) $$n_{i}\;\to \;n_{j}:\;{\left\{{\left\{M\right\}}_{k_{s}}\right\}}_{k_{ni}}\;then\;at\;{n_{j}:\;}_{:}{\left\{{\left\{{\left\{M\right\}}_{k_{s}}\right\}}_{k_{ni}}\right\}}_{k_{ni}}\;then\;n_{j}\;\to \;n_{k}:\;{\left\{{\left\{M\right\}}_{k_{s}}\right\}}_{k_{nj}}\;etc\ldots$$

#### 3.5.2. Key Pre-Distribution

In this scheme, the initial process is offline, such as the establishment of the authenticated neighbors list and the pre-distribution of keys. Each ordinary node has its own symmetric adjacent key. This key is shared only with a trustworthy neighbor in the line network topology. All ordinary nodes and the leader node must share a master key called the Leader node key, ‘$k_{Ln}$’. This key is used for all confidential communications between network members in processes such as when a new member joins the network, and for monitoring the behavior of the ordinary nodes. The individual key, ‘$k_{j}$’, is a unique pre-distributed key between every ordinary node and the leader node. This key is used in the re-keying phase during the revocation process.

#### 3.5.3. Re-Keying

In the case of a network member being revoked, only the leader node key, ‘$k_{Ln}$’, and one adjacent key needs to be renewed. Subsequently, every node in the network has a key re-generation mechanism to create a new key. This mechanism relies upon the Message Digest 5 algorithm (MD5) outlined in Table 5 and a hash of the Real Time Clock (RTC) value as shown below:

(2) $$k_{ni}\;=H\left(RTC\right)$$

Due to the straight line network topology, only the node located before the revoked node must update its own adjacent key, ‘$k_{nj}$’. This node then needs to share its new key with the new neighbor that replaces the revoked node in the authenticated neighbors list. This stage is coordinated by the *L_n_* via a method of unicasting a revoked message that is encrypted with a pre-distributed individual key, ‘$k_{j}$’. The revoked message contains elements such as the revoked node ID, the new neighbor ID and the new leader node key.

(3) $$\left\{New\_K_{Ln},ID_{j},ID_{k},{\mathrm{STAMP}}_{II}\right\}K_{i}$$

where ‘$New\_K_{Ln}$’ is the new leader node key, ‘$ID_{j}$’ and ‘$ID_{k}$’ are the identification numbers of the revoked node and the new authenticated neighbor respectively and the ‘${\mathrm{STAMP}}_{II}$’ part, which indicates the order of the elements in the revoked message as well as the length and hash of ‘$New\_K_{Ln}$’. The next step after receiving the revoked message is that the node will update its adjacent key, ‘$New\_k_{nj}$’, using its key re-generation mechanism and it shares this key with its authenticated new neighbor node:

(4) $${\left\{{New}_{K_{nj}}\right\}}_{{New}_{K_{LN}}}$$

**Figure 6:**
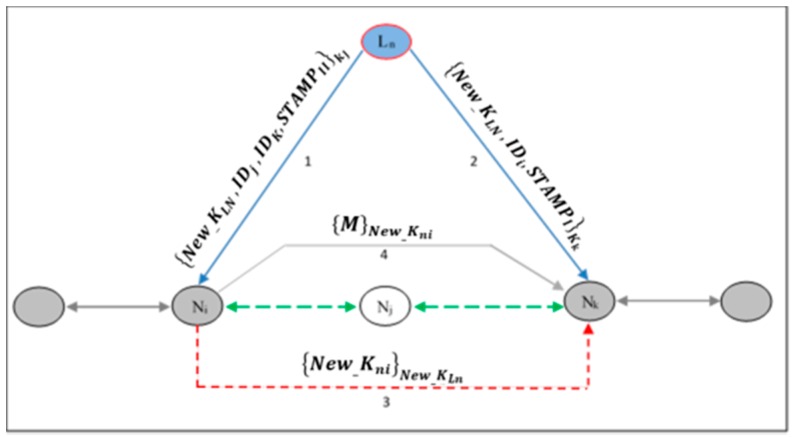
Network reconnection process.

The authentication process between new neighbors is coordinated by the leader node, as shown in Figure 6. Initially, the *L_n_* will send the authentication message of the new neighbor to the node that is located after the revoked node in the network topology. This message contains the new neighbor ID, the new leader node key and the ‘$STAMP_{I}$’ part, which indicates the order of the elements in the message as well as the length and hash of ‘$New\_K_{Ln}$’. Then, the new neighbors will establish a secured encrypted link:

(5) $${\left\{New\_K_{Ln},ID_{i},{\mathrm{STAMP}}_{I}\right\}}_{K_{k}}$$

#### 3.5.4. Memory Requirement

One of the major challenges in the establishment of a high security system in WSN is the limitation of memory capacity and storage. The constructed scheme occupies only a small memory size and this makes it suitable due to the limited storage capacity in sensor nodes. The binary sketch size of the uploaded program and the bootloader program stored in the Flash memory is 57,230 bytes of a maximum of 122,888 bytes available, and 4987 bytes of chip memory SRAM of a maximum of 8192 bytes available.

**Figure 7:**
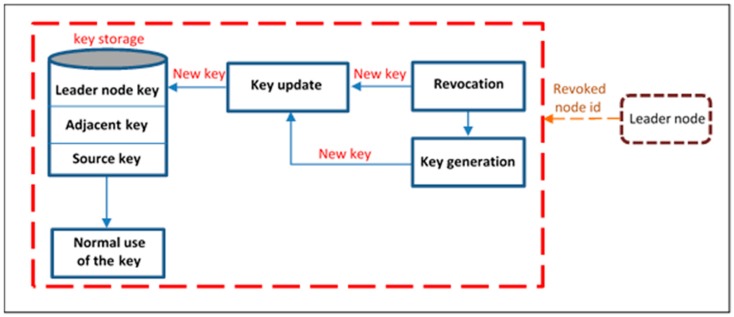
Keys administration.

Figure 7 shows the key administration operation in our scheme. If the leader node revokes any node member from the network, the key update phase will be performed by two of the revoked node neighbors in the line topology.

## 4. Practical Implementation of Proposed Framework

**Figure 8:**
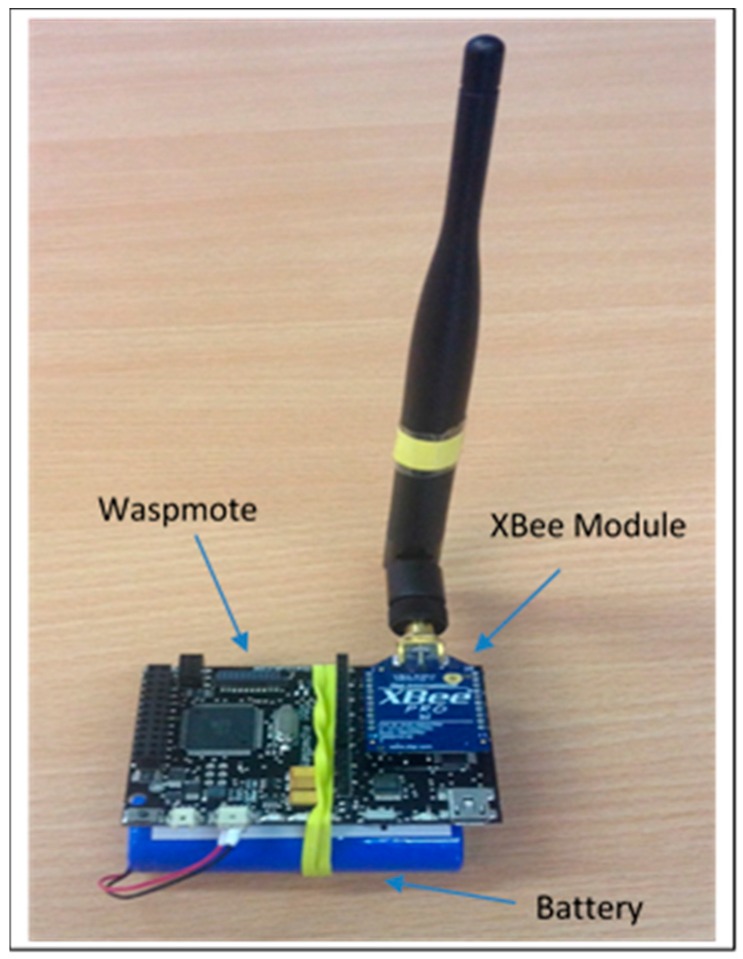
Waspmote platform integrated with XBee-pro module.

In this section, we present the implementation and test measurements of the scheme. The implementation involves four Waspmote nodes, a Waspmote Gateway, four XBee 802.15.4 Pro modules with antennae (Figure 8), a MC1322x USB ZigBee dongle, an Agilent 66321D Mobile communication DC Source, a Waspmote Pro IDE version 04 with Waspmote Pro API Version 013 software based on Arduino, X-CTU provided by Digi and the Wireshark network analyzer.

Below is an outline of the three scenarios used to provide measurements to obtain the optimum configuration. The three scenarios were:

(1) Four nodes and the gateway at a distance of 80, 120 or 160 m between end points in line topology.
(2) Three nodes and the gateway at a distance of 80, 120 or 160 m between end points in the line topology.
(3) One repeater node between sender and the gateway at a distance of 80, 120 or 160 m between end points in the line topology.

**Figure 9:**
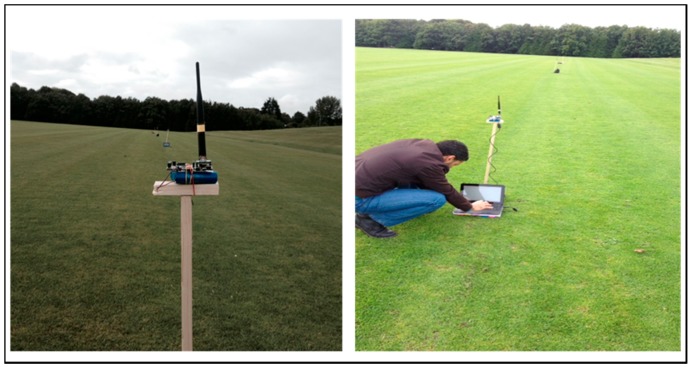
System outdoor deployment.

Figure 9 shows the physical setup of the scheme, where each Waspmote was placed on a fixed pole at a height of 80 cm from the ground. The effects of temperature and humidity on RSSI in WSNs as in [18] was considered. In this scenario the temperature was between 20 °C and 21 °C and the humidity was 70%.

### 4.1. Received Single Strength Indicator Measurement

**Figure 10:**
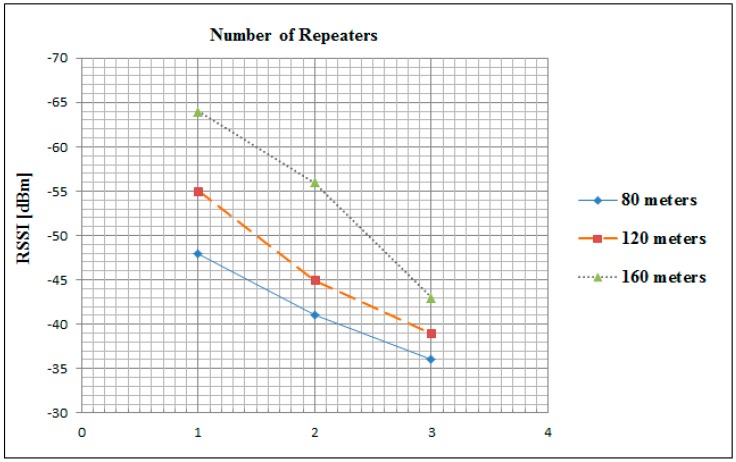
Average RSSI in three different scenarios of outdoor implementation.

In order to ascertain the received single strength indicator (RSSI) a test involving the transmission of a fixed amount of data was performed. In this experiment, the received single strength indicator (RSSI) was measured at the gateway. Figure 10 depicts the average value of RSSI which was measured after receiving 300 encrypted packets of 79 bytes in size at a baud rate of 115,200 bps.

These values were measured in the three aforementioned scenarios of distances and repeaters. The signal strength in the 80 m scenario was the strongest in the all case of one, two and three repeaters at exactly −48, −41 and −36 dB respectively. However, in the case of one repeater the 160 m scenario had the minimum signal strength value at −64 dB. While, in the case of all possible scenarios (as mentioned in Section 3) the maximum achievable signal strength is −36 dB, which is the 80 m distance with three repeaters scenario. These measurements are used by the leader node to determine the positions of future new joining nodes into the line topology.

### 4.2. Round Time Trip Measurement

**Figure 11:**
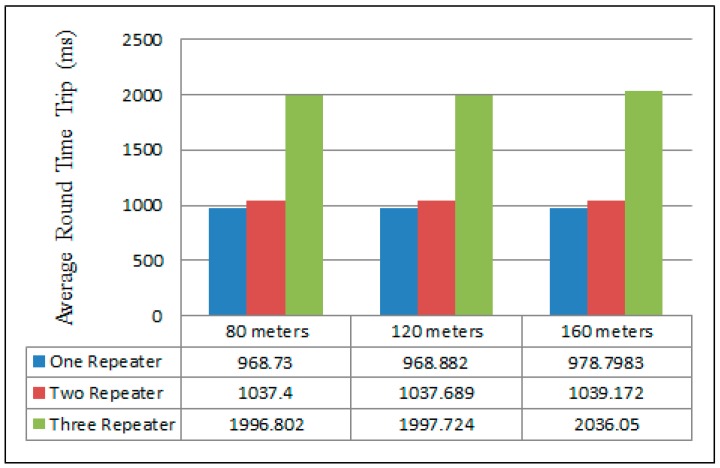
Measurements of RTT in three different scenarios at three different distances.

Figure 11 illustrates the average round time trip (RTT) for the different numbers of repeaters when increasing the distances between the sender and the gateway. In this experiment, RTT represents the elapsed time between the sender and the returned acknowledgment of the gateway to the last repeater. In fact, there was not a big difference in RTT measurements in the cases of one and two repeaters in all scenarios. However, in the three repeaters case, the time delay between 80 m and 160 m scenarios increased by approximately 39.248 ms, where RTT only increased by approximately 1.772 ms in the two repeaters case. The best RTT was obtained for the 80 m separation distance in all scenarios at 968.73, 1037.4 and 1996.802 ms in the one, two, and three repeater cases, respectively. These results were captured using a MC1322x USB Zigbee Dongle and the Wireshark network analyzer.

### 4.3. Current Consumption

**Figure 12:**
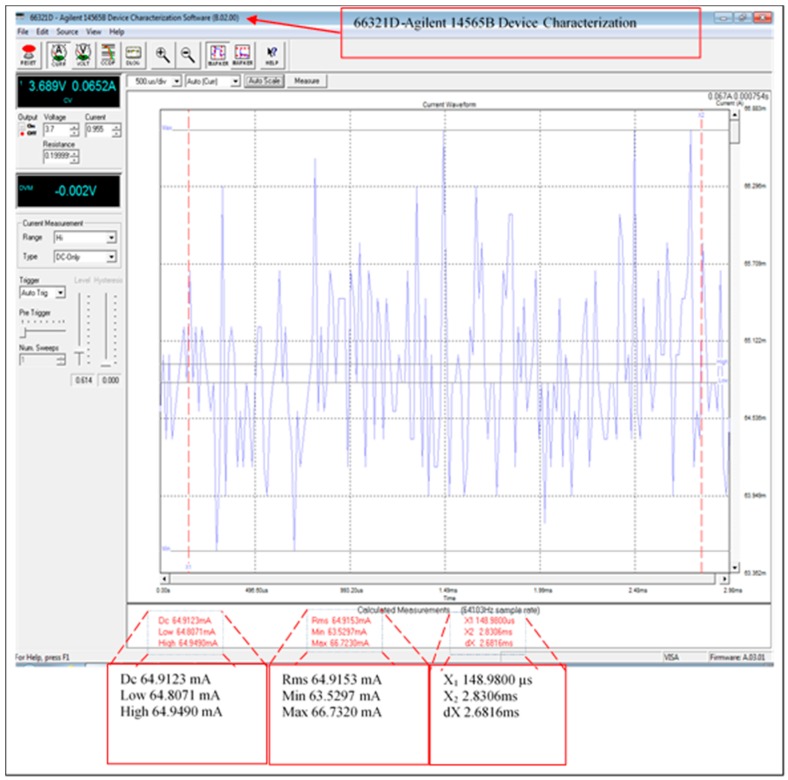
Fully operational average current consumption.

Data processing relies on the size of the data and the approach used in processing this data. Furthermore, the designed scheme has been adapted to use minimum current consumption for data processing. Figure 12 illustrates the average current consumption of the fully functional scheme including the XBee transmission module current consumption. However, the current consumption of the transmission data has been improved by using sleeping schedules for the receiving/transmitting modules (the XBee current consumption is from 37 to 64 mA with the mode ON fully operational). The measurements were taken using the 66321D-Agilent with input 3.689 V and 0.19999 Ω resistance. The average current consumption of the XBee module is 64.9123 mA when the leader node is fully operational, this was significantly improved by introducing the sleeping mode feature as described below.

**Figure 13:**
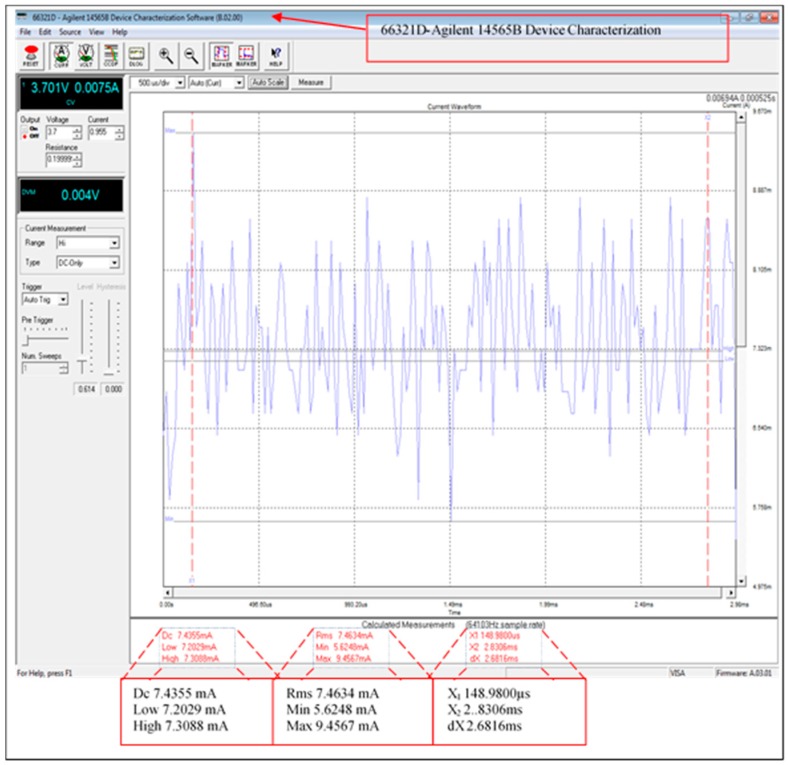
Average current consumption of sleeping mode phase.

The XBee module in the leader node will return to sleeping mode after transmitting/receiving data. Figure 13 shows the average current consumption of the sleeping mode of the XBee modules in the scheme. Where this is the total current consumption during data processing except for the transmission/receiving phase. The measured current consumption has been improved from 64.9123 to 7.4355 mA, which is a reduction of over 88%. Table 6 provides the current consumption of the scheme modes, this includes when the XBee module is fully functional and sleeping.

## 5. Discussion and Comparisons

**Figure 14:**
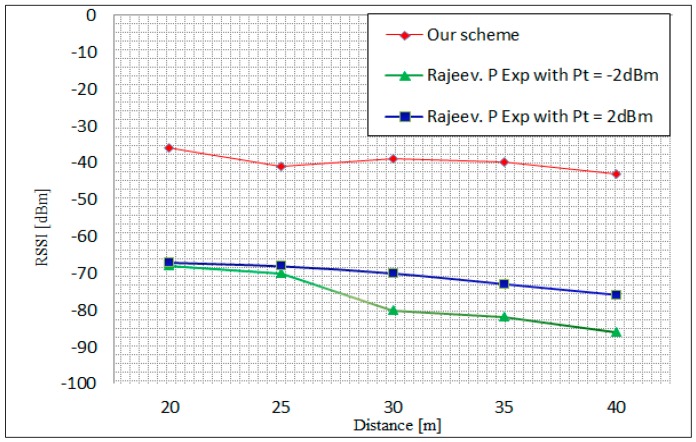
Proposed scheme measured RSSI values with different distances versus the Piyare [19] results.

In [19] Piyare et al. evaluated the performance of ZigBee networks based on XBee modules in terms of RSSI. The experiment was based on the single-hop and multi-hop in line network topology. In this experiment, average values of the RSSI was measured after transmitting 50 packets of 30 Bytes with varied distance between the sender and the receiver. Figure 14 illustrates the relationship between the measured RSSI and the distance using two different transmit power values of −2 dBm and 2 dBm. In our scheme, the measurements have been taken after transmitting 300 encrypted packets of 97 Bytes in an outdoor environment. As shown in Figure 14, the three measured values of RSSI decreased when the distance between the sender and the receiver was increased. However, our scheme presents strong signal strength values at all distance scenarios, where the strongest value is −36 dBm at 20 m and the weakest value was −43 dBm at 40 m. In the 20 and 30 m distances, the fluctuation in this scheme is graphed and can be correlated with interference from other networks, e.g., reflection phenomena.

**Figure 15:**
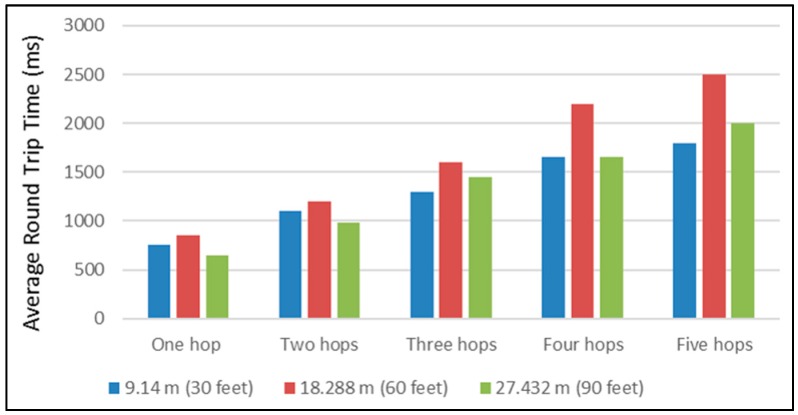
Multi-hop measurement of average round trip time [12].

**Figure 16:**
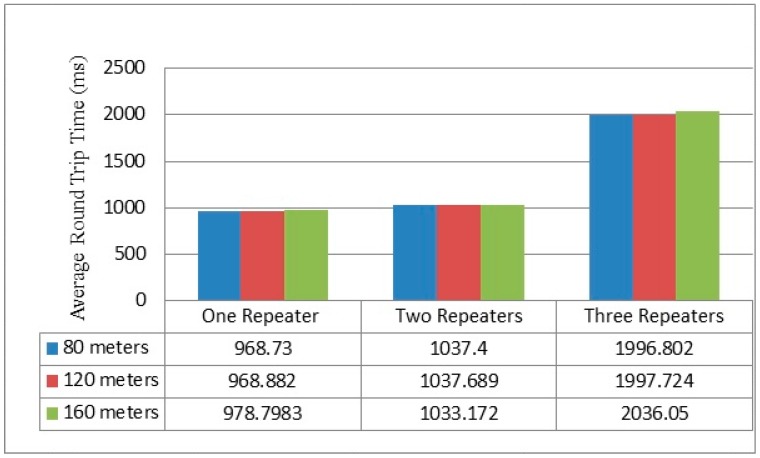
Multi-repeaters round trip time measurement result.

When comparing the work undertaken, it was compared to Jorg and Guangyu [12]. Figure 15 presents their result of the average Round Trip Time RTT of line network topology. The experimental setup consisted of multiple different scenarios of distance and number of repeaters. Initially, the scenarios were: 9.14 m (30 feet), 18.288 m (60 feet), and 27.432 m (90 feet) between nodes, with one hop, two hops, three hops, four hops, and five hop scenarios (one hop per repeater). Furthermore, Figure 16 depicts the three scenarios of our scheme, which are one repeater, two repeaters, and three repeaters in 80, 120 and 160 m distances between the sender and the gateway.

In the cases of 40 and 60 m distances between the nodes in the single repeater scenario, both schemes presented approximately the same time delay. However, in the two repeaters scenario, at a distance of 55 m the setup showed a time delay of 1033.172 ms and [12] presents approximately 1300 ms. In addition, the time delay between our scheme and [12] was around 170 ms in the same scenario at the distance of 27 m. Comparatively, travelling packets in the scheme being presented needed larger delays in comparison with [12] at distance of 40 m in the case of the three repeaters scenario. Generally, the time of receiving transmitting packets increase with the number of repeaters.

In Table 7 and Table 8, a comprehensive comparison between the scheme presented and three other schemes in terms of cryptographic scheme, size and number of stored security keys, memory space used by schemes, maintenance and re-keying strategy, scheme implementation environment, nodes and coordination, is shown.

## 6. Conclusions

This paper presents an analysis of the security performance of a WSN running a symmetric encryption security scheme that is suitable for use in a maritime coastal environment. The work made the re-keying process a local operation and minimized the use of key memory storage. The technique was implemented on the Waspmote platform and analyzed in terms of a received single strength indicator (RSSI), average round time trip (RTT), and current consumption. These measurements were taken in real time at the University of Limerick campus. The scheme implemented has improved some common security issues in WSNs, including:

- *Efficiency*: 88.55% of current consumption was reduced using the XBee sleeping mode. This will improve the lifetime of all network sensor nodes.
- *Data confidentiality*: by doubling the encryption of the messages, we ensure that only the gateway in the network can decrypt the original data (using AES 128) and after that, we establish Peer-to-Peer encryption between repeaters.
- *Authentication*: each node has an individual key shared with the leader node, which is used to ‘sign’ the messages in order to ensure the authenticity of the new neighbor and the rekeying. This signing is performed when the leader node reconfigures the network topology in the case of joining or revoking network members.
- *Memory capacity storage*: by using the re-generation function, there is no need for each node to store as many keys as are required in some key pre-distribution schemes where a large pool of keys is located in each node before deployment [20]. In our scheme, each node has only four keys. Therefore, every node requires only a small amount of memory for key storage, which is four times 128 bits.
- *Non-repudiation*: by signing the XBee acknowledgment messages, there is now verifiable proof that the information sent has really been received by a specific sensor node. This signing using a shared source/destination key, *K_s_*, provides weak non-repudiation as a public key scheme is not yet available in our system.
- *Forward security:* in the reconfiguration of the scheme, new neighbors could not communicate directly before authentication process. The authentication process coordinates via a leader node.
- *Backward security*: when a node is revoked from the network, the system ensures that the node cannot receive any new data from the network.

This work is progressing at our center and the current focus of the work is to facilitate the implementation of an efficient public key scheme for use in the protocol. The availability of an efficient public key scheme will enable the generation of digital signatures, provide strong non-repudiation and will further enhance key distribution for use in a line topology network [21,22,23,24].

## Figures and Tables

**Table 1:** Explanation of notation used in Figure 1.

Symbol	Description
*n_i_*, *n_j_*, *n_k_*, *n*_l_	Ordinary nodes in the line topology
*L_n_*	Leader node
{ }*_k_*	Symmetric encryption/decryption with key *k*
*M*	Transmitted sensor data
*k_s_*	Secret encryption source key
*k_ni_*	Secret encryption adjacent key of node *i*
*k_i_*	Secret encryption individual key of node *i*
*k_Ln_*	Secret encryption leader node key
{{*M*}*_ks_*}*k_ni_*	Sensor data encrypted with source and adjacent keys
{*M*}*k_i_*	Message encrypted with individual key of node *i*
{*M*}*k_Ln_*	Message encrypted with leader nod key

**Table 2:** Modules integrated in Waspmote.

Module	Protocol	Frequency	Tx_Power	Sensitivity	Range
Xbee 802.14.5-pro	802.14.5	2.4 GHz	100 mW	−100 dBm	7000 m
XBee ZBee-Pro	ZBee-Pro	2.4 GHz	50 mW	−102 dBm	7000 m
XBee 868	RF	868 HMz	315 mW	−112 dBm	12 km
XBee 900	RF	900 MHz	50 mW	−100 dBm	10 km
WiFi	802.11b/g	2.4 GHz	0–12 dBm	−83 dBm	7000 m
GPRS	-	8500/900/1800/1900 MHz	2W(Class 4) 859/900 MHz 1W(Class 1) 1800/1900 Mhz	−109 dBm	km typical carrier range
3G/GPRS	-	Tri-Band UMS 2100/1900/900 MHz Quad-based GSM/EDGE, 850/900/1800/1900 MHz		−106 dBm	km typical carrier range
Bluetooth Low Energy	Bluetooth v.4.0/Bluetooth Smart	2.4 GHz		−103 dBm	100 m

**Table 3:** Maximum frame size per protocol.

Module			Maximum Frame Size
XBee-802.15.4	Link Encrypted	@16bit Unicast	98 Bytes
		@64bit Unicast	94 Bytes
		Broadcast	95 Bytes
	Link Unencrypted		100 Bytes
XBee-868			100 Bytes
XBee-900	Link Encrypted		80 Bytes
	Link Unencrypted		100 Bytes
XBee-Digimesh			73 Bytes
XBee-ZigBee	Link Encrypted	@64bit Unicast	66 Bytes
		Broadcast	84 Bytes
	Link Unencrypted	@64bit Unicast	74 Bytes
		Broadcast	92 Bytes
Bluetooth-transparent connection			Limited by MAX_FRAME
GPRS			Limited by MAX_FRAME
3G			Limited by MAX_FRAME
LoRa/SX1272			Limited by MAX_FRAME
WiFi			Limited by MAX_FRAME

**Table 4:** AES-128 with ECB cipher mode and zeros padding.

Algorithm	Key Size	Data Block Size	Mode Cipher	Padding
AES-128	128 bits	16 Bytes	ECB	ZEROS

**Table 5:** MD5 hash algorithm.

Algorithm	Output Size (Bits)	Internal State Size (Bits)	Block Size (Bits)	Max Message Size (Bits)	Word Size (Bit)
MD5	128	128	512	2^64^ − 1	32

**Table 6:** Measured current consumption of scheme modes.

Scheme Mode	Measured Current Consumption (mA)		
	Max	Min	Average
Fully functional system	66.732	63.5297	64.9123
XBee module (sleeping)	9.4567	5.6248	7.4355
XBee module current consumption (awake)	57.2753	57.9049	57.4768

**Table 7:** Comparison between schemes.

Scheme	Offline Phase	Cryptographic Scheme	Key Size (bits)	Communication Type	Number of Storage Keys	Memory Space Used by the Scheme	Re-Keying Strategy
Neighborhood Scheme [12]	Certificate stored in each node	Hybrid (RSA and symmetric)	128	Multicast/Unicast	Each node stores: own public/private keys; own secret neighborhood key; neighbors secret key(s), and source key (used when the node is data source)	Not Given	Local operation, where each node updates keys with its current neighbor.
Hierarchical Key Management Scheme [13]	No pre-loaded keys	Hybrid (D–H and Symmetric)	Not Given	Broadcast (2-hop adjacent node)	Level 1 head stores its secret key and secret key of Level 2 head. Level 2 head stores its secret key and secret key of Level 1 head. Ordinary node stores the secret key of the Level 2 head, secret communication key, and DH keys.	Not Given	Complicated operation where each level head node must regenerate a number of keys.
Time-Based Key Management Scheme [15]	Keys pre-loaded	Symmetric	64	Broadcast	500 keys in center node. 100 keys for sensor nodes (to ensure sharing of a key with at least one of 10 neighbors)	4 KB for center node 0.8 KB for sensor node	Only the nodes deployed at the same time when compromised node is revoked have to regenerate keys.
Presented Scheme	Keys pre-loaded	Symmetric AES-128 in ECB mode with zeros padding. Key generation -MD5 and RTC.	128	Unicast	(5 keys) Each ordinary node stores; own adjacent key; neighbor shared adjacent key; an individual key that is shared with leader node; Leader node master key, and a source key (used when the node is data source).	Program size 57,230 bytes Ram required 4987 bytes.	Re-keying in this scheme is a local operation, where only one node must update its adjacent key, in addition to the leader node key.

**Table 8:** Comparison between schemes.

Scheme	Implementation Environment	Node Coordination	Comments
Neighborhood Scheme [12]	Simulation using the Glomosim Simulator. Used HP iPAQ 550 PDAs outdoors.	Node authentication is performed without coordination with other nodes.	Provides protection against attacks to the routing protocol using X509, RSA and a sequence number. Rekeying is a local operation between the nodes that share neighborhood keys with a revoked node. Provides forward and backward security. Does not rely on an online trusted third party.
Hierarchical Key Management Scheme [13]	Simulation only	Level 1 head coordinates all Level 2 heads in its subgroup. Level 2 head coordinates all ordinary heads in its subgroup.	Uses a spanning tree topology in ad hoc networks. Provides the forward and backward security. Latency issues during the authentication process and updating secret keys.
Time-Based Key Management Scheme [15]	Simulation only	For additional node deployment a centre node coordinates the authentication.	The scheme is not suitable for a large sensor network because of key storage requirements. Offers protection against wormhole and sinkhole attack. Provides forward and backward security.
Presented Scheme	Waspmote nodes and Gateway, XBee 802.15.4 Pro module with antenna. MC1322x USB ZigBee dongle. Agilent 66321D Mobile DC Source. Waspmote Pro IDE v04 with Pro API v013 software based on Arduino. X-CTU provided by Digi and the Wireshark network analyzer in an outdoor implementation.	The Leader node monitors the behaviour of all ordinary nodes in this scheme. It is responsible for the authentication, revocation, and reconfiguration phases of other nodes.	Provides protection against attacks to the routing protocol using location based routing. Node addition or revocation is handled by the Leader Node. Rekeying is a local operation between the nodes that share neighborhood keys with a revoked node. Provides forward and backward security. Does not rely on online trusted third party. All message exchanges are acknowledged.
